# Modulation of platelet functions by crude rice (*Oryza sativa*) bran policosanol extract

**DOI:** 10.1186/s12906-016-1223-9

**Published:** 2016-07-28

**Authors:** Wai-Teng Wong, Maznah Ismail, Mustapha Umar Imam, Yi-Da Zhang

**Affiliations:** 1 Laboratory of Molecular Biomedicine, Institute of Bioscience, Universiti Putra Malaysia, 43400 Serdang, Selangor Malaysia; 2 Department of Nutrition and Dietetics, Faculty of Medicine and Health Sciences, Universiti Putra Malaysia, 43400 Serdang, Selangor Malaysia; 3 Precision Nutrition Innovation Institute, College of Public Health, Zhengzhou University, Zhengzhou, 450001 Henan Province China; 4 Cardiology Department, Affiliated Hospital of Chengde Medical University, 067000 Chengde, Hebei China

**Keywords:** Arachidonic acid, Adenosine diphosphate, Collagen, Platelet aggregation, Platelet adhesion, Laminin, Protein secretion

## Abstract

**Background:**

Rice bran is bioactive-rich and has proven health benefits for humans. Moreover, its source, the brown rice has antioxidant, hypolipidemic and other functional properties that are increasingly making it a nutritional staple especially in Asian countries. This study investigated the antiplatelet aggregation mechanisms of crude hexane/methanolic rice bran extract, in which policosanol was the targeted bioactive. Platelets play a vital role in pathogenesis of atherosclerosis and cardiovascular diseases, and their increased activities could potentially cause arterial thrombus formation or severe bleeding disorders. Thus, in this study, platelet aggregation and adhesion of platelets to major components of basal lamina were examined in vitro*.* In addition, cellular protein secretion was quantified as a measurement of platelet activation.

**Methods:**

Adenosine diphosphate (ADP), collagen, and arachidonic acid (AA)-induced aggregation were studied using the microtiter technique. Rat platelets were pre-treated with various concentrations of policosanol extract, and the adhesion of platelets onto collagen- and laminin-coated surface (extracellular matrix) was studied using the acid phosphatase assay. The effect of crude policosanol extract on released proteins from activated platelets was measured using modified Lowry determination method.

**Results:**

Rice bran policosanol extract significantly inhibited in vitro platelet aggregation induced by different agonists in a dose dependent manner. The IC_50_ of ADP-, collagen-, and AA-induced platelet aggregation were 533.37 ± 112.16, 635.94 ± 78.45 and 693.86 ± 70.57 μg/mL, respectively. The present study showed that crude rice bran policosanol extract significantly inhibited platelet adhesion to collagen in a dose dependent manner. Conversely, at a low concentration of 15.625 μg/mL, the extract significantly inhibited platelet adhesion to laminin stimulated by different platelet agonists. In addition to the alteration of cell adhesive properties, cellular protein secretion of the treated platelets towards different stimulants were decreased upon crude extract treatment.

**Conclusion:**

Our results showed that crude rice bran policosanol extract could inhibit in vitro platelet adhesion, aggregation and secretion upon activation using agonists. These findings serve as a scientific platform to further explore alternative therapies in cardiovascular diseases related to platelet malfunction.

## Background

The platelet is the smallest blood cell, which plays a major role in the occurrence of thrombosis, and to a greater extent, cardiovascular diseases. Stroke and coronary artery diseases are partly attributed to improper platelet activation [1]. Hyperlipidemia, diabetes, hypertension, poor dietary and living patterns, as well as genetic inheritance are factors that promote platelet hyperactivity [2–5]. The critical balance of pro-coagulants and anti-coagulants in biological systems is crucial in preventing haemostatic complications [6]. Platelet activation sets in motion a cascade of events initiated by adhesion to exposed endothelial tissue (attachment to basal lamina), morphologic changes (expression of surface glycoproteins), cellular secretion (dense and alpha granule contents), and finally platelet aggregation. These eventually result in development of strong thrombus and atherosclerotic plaques through platelet-leucocytes interaction [7, 8].

Regulation of platelet functions using pharmacological agents or drugs such as aspirin, clopidogrel, or ticlopidine is an effective approach for the prevention of thrombotic plaques and atherosclerosis [9]. However, in recent decades, public perception on diet is changing, with an increasing number of people believing that natural foods that contain bioactive constituents could possibly diminish the risk factors of cardiovascular continuum [10–13]. Bioactives present in different fruits and vegetables are reported to be useful and good candidates as antiplatelet agents from natural-based or herb-based products as alternatives to commercial antiplatelet drugs [14–16].

Rice bran, a valuable source of food bioactives, is under-valued and under-utilized for applications until now [17]. Rice bran contains essential fatty acids, proteins, dietary fibers, vitamins, oil and other constituents, and is long believed to have health benefits particularly in cardiovascular diseases [18]. Rice bran is reported to have cholesterol-lowering properties in hypercholesterolemic hamsters and humans [19, 20]. In addition to that, rice bran was shown promisingly to have blood glucose-lowering, chemo-preventive and anti-aging properties [21–23]. Policosanols, which are long chain aliphatic fatty alcohols, are well acknowledged for their positive influence in pathophysiology. Previous studies had shown that sugarcane policosanol inhibited platelet aggregation by reducing serum thromboxane A_2_ yet increasing prostacyclin levels in rodents [24–26]. Furthermore, 200 mg/kg of sugarcane policosanol treatment significantly protected Mongolian gerbils from ligated carotid artery-induced cerebral ischemia, and this was also suggested to be a result of reduction in serum thromboxane A_2_ levels and increment in prostacyclin [27]. D-003, long chain aliphatic fatty acids which are structurally and metabolically closely related to the corresponding fatty alcohols, was shown to inhibit ex vivo collagen and adenoside diphosphate- (ADP-) induced aggregation effectively in rats. The mechanisms could be associated with prostaglandin synthesis and the protective effect against lipid peroxidation [28, 29].

Taking into account the above-mentioned, the present study was undertaken to determine the antiplatelet function of crude rice bran policosanol extract as there was no clear cut information on the mechanisms of action of rice bran policosanol although antiplatelet effects of other rice bran extracts were reported [30–32]. According to Cicero and Derosa [33], unlike other sources of policosanol, there are no studies currently demonstrating the antiplatelet effects of rice bran-derived policosanol. In regards, this paper described the effects of crude rice bran hexane/methanolic extract on rat platelet functions covering platelet adhesion to different coated surfaces, granular protein secretion and platelet aggregation towards different platelet activators.

## Methods

### Materials

Laminin, bovine serum albumin (BSA), p-nitrophenyl phosphate, and Triton X-100 were purchased from Merck (Darmstadt, Germany). Type I rat tail collagen was purchased from Sigma Aldrich Co. (St. Louis, MO, USA). Arachidonic acid (AA), equine tendon collagen, and ADP were purchased from Chrono-log Corp (Havertown, PA, USA). All other reagents were of analytical grade and were provided by commercial suppliers.

### Analytical procedures

#### Plant materials

Rice bran was obtained from Bernas milling factory in Kuala Selangor, Selangor, Malaysia. Rice bran sample with size of 4 mm was stabilized by heat treatment using an automated microwave oven (Microwave conditions: 2450 MHz, 550 W, 110 °C, 200 s). The sample was stored in 4 °C during the whole analysis.

### Extraction

Policosanol was extracted using solid–liquid extraction according to the described procedures with minor modifications [34]. Briefly, 10 g of rice bran were placed in glass flask with approximately 150 mL of a mixture of hexane and methanol (20:1 v/v). Extraction was performed by sonication technology (50Hz, 350 W, 50 °C, 3 h) using Power Sonic 505 ultrasonicator (Hwashin Technology Co., Seoul, Korea). The rice bran residues were removed from the solvent extract by centrifuging at 4000 rpm for 10 min. The solvent was completely removed from the extract using a rotary-evaporator under vacuum at 40 °C leading to greenish-yellow extract.

### GCMS analysis of policosanol extract

The fatty alcohol was transformed into trimethylsilyl ethers using N, O-Bis (trimethylsilyl) trifluoroacetamide. The samples and standards were derivatized by incubating at 60 °C for 20 min and subsequently analyzed using gas chromatography mass spectrophotometry (TSQ series; Thermo Scientific, Waltham, MA, USA). Determination of policosanol content was done according to the method described by Ishaka et al. [35] with minor modifications. Standard mixture was prepared using chloroform, and 500 μL of this mixture was derivatized with 200 μL of derivatizing agent, after which the volume was made up to 1 mL by chloroform after cooling to room temperature. The GC oven temperature was programmed from 150 to 300 °C with a heating rate of 4 °C/min and maintained at this temperature for 15 min. Initial flow rate of the carrier gas, helium, was 1.0 mL/min, while the inlet temperature was 300 °C. GC-MS parameters were as follows: the MS transfer line temperature was 280 °C, the ion source was kept at 230 °C, and the MS quadrupole temperature was kept at 150 °C. The ionization energy was 70 eV with 2 scans/s and a mass range of 100–1000 amu. The standards/samples (2 μL) were injected into GC-MS with a 1:10 split ratio.

### Ethics approval and animal handling

Rat blood was obtained from Sprague–Dawley species with the ethical approval from institutional animal care and use committee (IACUC), Faculty of Medicine and Health Sciences, Universiti Putra Malaysia. Male rats were acclimatized at the animal house (25–28 °C) for one week with *ad libitum* rodent chow and free access to clean pipe water.

### Blood platelet isolation

Platelet isolation was performed according to the described procedures with minor modifications [36]. Blood was collected using tri-sodium citrated tubes (109 mM 3.2 %). Platelet rich plasma (PRP) was isolated by centrifugation at 100 × g for 20 min. PRP was then centrifuged for additional 10 min at 1400 × g to sediment platelet pellet. The pellet was suspended in Tyrode HEPES buffer (134 mM NaCl, 2.9 mM KCl, 0.34 mM Na_2_HPO_4_, 12 mM NaHCO_3_, 20 mM HEPES and 5 mM glucose, pH 7.3), and washed twice to remove other cellular debris. Prostacyclin (50 ng/mL) was added during platelet isolation and washing steps. The platelets were suspended in Tyrode HEPES buffer at a final concentration of 10^9^cells/mL.

### Platelet aggregation assay

Platelet aggregation was studied using microtiter plate according to the described procedures with minor modifications [37]. Policosanol extract was dissolved in DMSO prior to all tests. Then, 100 μL of platelet suspension pre-treated with extract (extract final concentration 125–1000 μg/mL, incubated for 10 min) was pipetted into 96 well plates. Agonists were added to the wells accordingly (final concentration of ADP, collagen, and AA was 10 μM, 5 μg/mL, and 0.5 mM respectively). Double orbital shaking mode was used and the optical density at 405 nm was read every one min for 20 min using BioTeK Synergy H1 Hybrid Reader (BioTek Instruments Inc., Winooski, VT, USA). Platelet aggregation was calculated by subtracting the final reading from the initial reading of the same well followed by normalization with the DMSO control.

### Platelet adhesion assay

Adhesion of platelets to laminin and collagen was determined according to the described procedures with minor modifications [38, 39]. Platelets were pre-incubated with policosanol extract at various concentrations (10 min at 37 °C) and DMSO served as vehicle control. Using a 96-well plate, 50 μL of 40 μg/mL of collagen (0.05 % in CH_3_COOH) or 50 μL of laminin solution (1 mg/mL in phosphate buffer solution) was pre-incubated for 2 h. The wells were subsequently treated with 200 μL of PBS containing 1 % BSA for 1 h after aspiration and washing with 200 μL of PBS. AA (0.5 mM), ADP (10 μM), and collagen (5 μg/mL) were used as platelet activators, and incubated with 50 μL of platelet suspension per each coated well at 37 °C for 1 h. The plate was washed at least three times with 200 μL PBS to remove unattached platelets. Subsequently, 140 μL of the substrate solution containing 1 mg/ml *p*-nitrophenyl phosphate in citrate buffer (0.1 M sodium citrate, 0.1 M acetic acid and 0.1 % (w/v) Triton X-100, pH 5.4) was added to each well. The reaction was stopped after 1 h incubation at 25 °C and the color was developed by addition of 100 μL of NaOH (2 N). The absorbance of the reaction product, *p*-nitrophenol, was measured at 405 nm using BioTeK Synergy H1 Hybrid Reader (BioTek Instruments Inc., Winooski, VT, USA).

### Platelet acid phosphatase assay

A calibration curve was used to relate the platelet numbers to their acid phosphatase activity. This was performed according to the described procedures [40]. For the estimation of the total platelet count, 50 μL of PRP was used, and for blank, 50 μL of platelet poor plasma (PPP) was used. Platelet suspensions containing known cell numbers were dispensed in uncoated wells and incubated for 60 min at 25 °C with substrate solution containing 1 mg/ml *p*-nitrophenyl phosphate in citrate buffer (0.1 M sodium citrate, 0.1 M acetic acid and 0.1 % (w/v) Triton X-100, pH 5.4). The reaction was stopped and the color was developed by addition of 100 μL of NaOH (2 N). Subsequently, the absorbance of the reaction product, *p*-nitrophenol, was measured at 405 nm using BioTeK Synergy H1 Hybrid Reader (BioTek Instruments Inc., Winooski, VT, USA). The constructed graph was used to determine the number of platelets attached to laminin- and collagen-coated plates.

### Protein secretion assay

Platelet suspension was incubated with policosanol extract at various concentrations (7.8125–1000 μg/mL) for 60 min at 37 °C. A standard curve was constructed using BSA powder. One hundred microliters (100 μL) of standards/samples were dispensed into wells and 200 μL of Biuret reagent was added to each well and mixed thoroughly. Biuret reagent was prepared by mixing 0.5 mL of 1 % cupric sulfate with 0.5 mL of 2 % sodium potassium tartrate, followed by the addition of 50 mL of 2 % sodium carbonate in 0.1 N NaOH. The mixture was incubated (15 min, 25 °C) followed by the addition of 20 μL 1.0 N Folin-Ciocalteu’s reagent into each well. Color was allowed to develop for 30 min at room temperature and the absorbance measured at 650 nm with BioTeK Synergy H1 Hybrid Reader (BioTek Instruments Inc., Winooski, VT, USA) [41].

### Statistical analysis

The data were analyzed using minitab 16 (Minitab Inc, State College, Pennsylvania, United States) by one-way analysis of variance (ANOVA) and presented as means ± standard deviation (SD). The significant differences between groups were determined at *p* < 0.05.

## Results and discussion

**Fig. 1:**
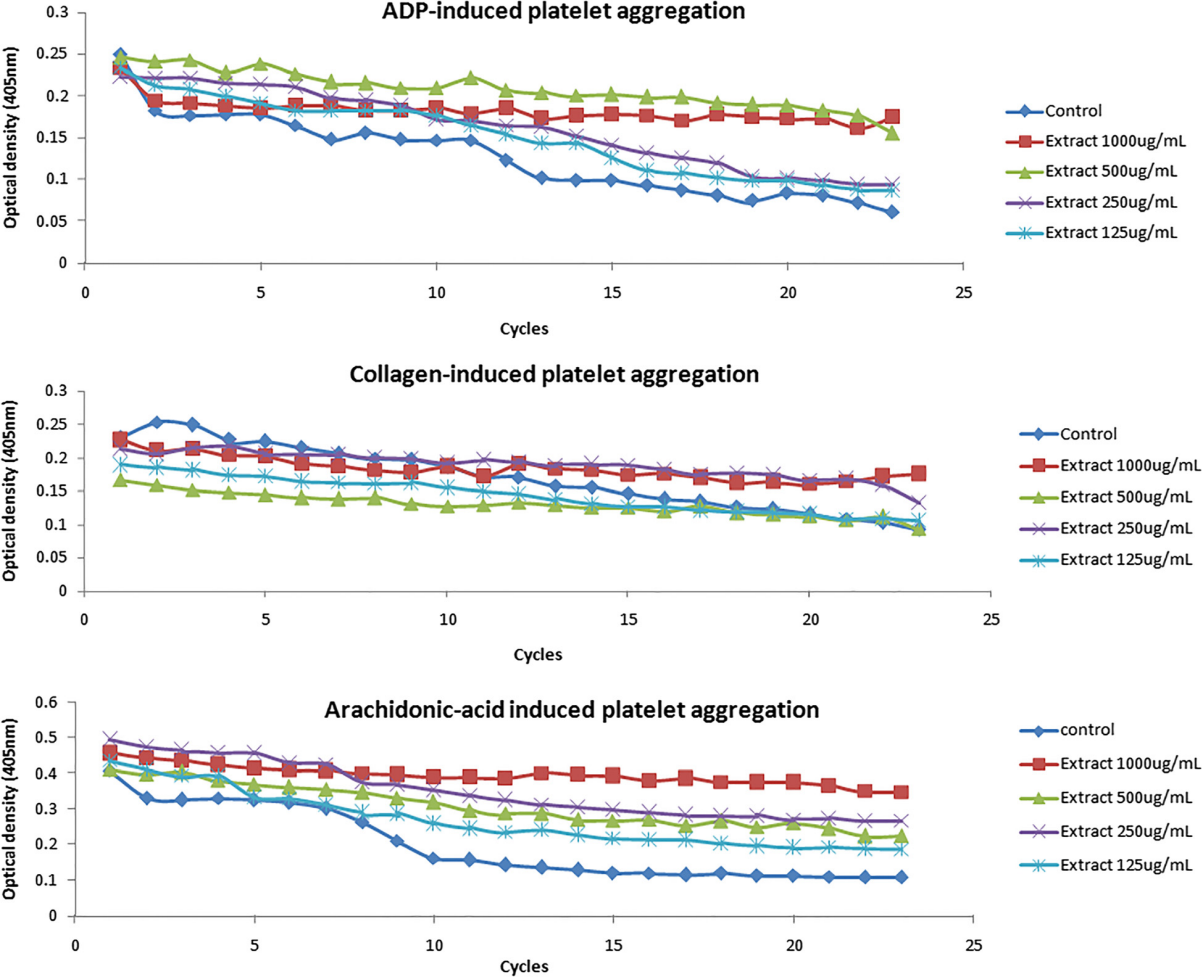
Platelet aggregation pattern obtained in double orbital shaking mode. 100 μL of platelet suspension was activated by platelet agonist (ADP, collagen, AA) upon treatment of rice bran extract dissolved in DMSO or vehicle control (DMSO). The aggregation trace was studied for 23 cycles with a fix time interval of one min

The present study demonstrated the effect of rice bran policosanol extract (*Oryza sativa*) on platelet function. From our unpublished data, policosanol content of the extract was reported as 877.99 ± 110.11 mg/100 g extract. Figure 1 showed the optical density trend for 25 min recorded using BioTeK Synergy H1 Hybrid Reader (BioTek Instruments Inc., Winooski, VT, USA). The optical density decreased with time as individual platelet cells were increasing becoming fewer. Optical density value decreased upon agonist addition and degree of platelet aggregation was calculated using the absorbance difference.

The present study showed that rice bran policosanol extract exerted antiplatelet aggregation effect towards ADP, collagen and AA as depicted in Table 1. From the tabulated data, we successfully demonstrated that policosanol extract inhibited platelet aggregation in a dose dependent manner. It was shown to have the strongest inhibitory action towards ADP-induced platelet aggregation. Low dose rice bran extract (125 μg/mL) significantly inhibited platelet aggregation induced with ADP, collagen, and AA, while at 1000 μg/mL, the extract significantly inhibited ADP, collagen, and AA-induced platelet aggregation by up to 67.36 ± 4.30 %, 62 ± 5.85 %, and 65.25 ± 4.27 %, respectively.

**Table 1:** Degree of platelet aggregation after treatment with extract

Extract (μg/mL)	Platelet aggregation (%) by different agonists		
	Adenosine diphosphate (10 μM)	Collagen (5 μg/mL)	Arachidonic acid (0.5 mM)
0 (control)	100	100	100
1000	32.64 ± 4.30	38.00 ± 5.85^a^	34.75 ± 4.27
500	47.21 ± 5.95^a^	45.56 ± 4.22^a^	60.58 ± 3.68^a^
250	56.98 ± 6.06^a^	66.29 ± 2.86^b^	67.23 ± 1.25^a,b^
125	73.61 ± 4.50	75.49 ± 2.16^b^	73.98 ± 1.31^b^
IC_50_ (μg/mL)	533.37 ± 112.16	635.94 ± 78.45	693.86 ± 70.57

The presented values were mean ± standard deviation. Means that shared the same letter in any column were not significant different at *p* < 0.05

**Fig. 2:**
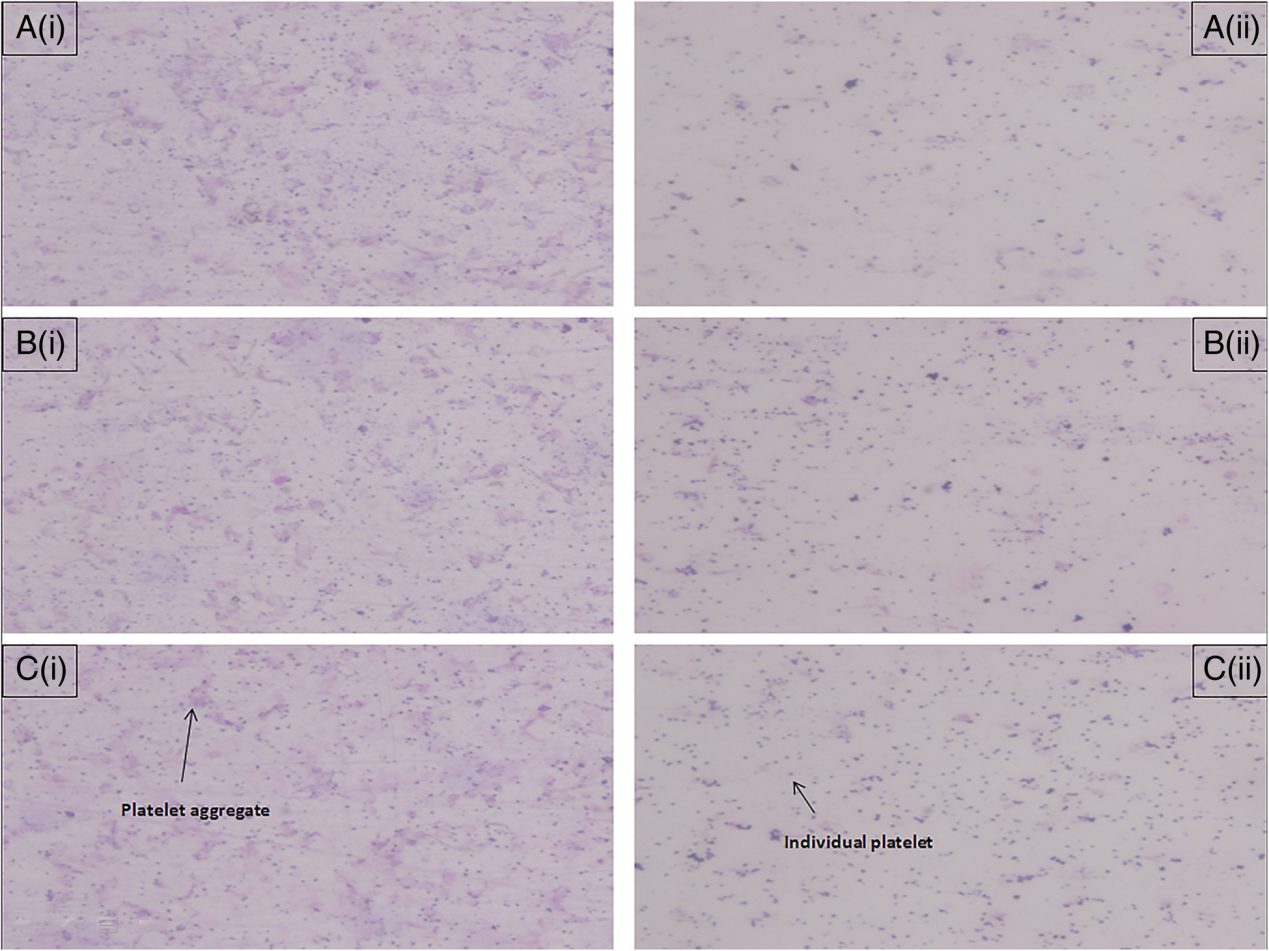
Platelet was fixed with 0.5 % formaldehyde after incubation with rice bran extracts. Smears were loaded on clean glass slide and covered with a cover glass. The samples were stained with Wright’s stain and air dried before subjected to microscopic observation (Olympus, Japan). Platelet was activated by ADP, AA, and collagen (**a**, **b**, **c**) upon treatment with (i) vehicle control (ii) rice bran extract (500 μg/mL) for 10 min at 37 °C

Furthermore, microscopic images of platelet aggregation are shown in Fig. 2. These observations again validated the antiplatelet effect of rice bran policosanol extract. The exact aggregation inhibitory mechanisms are yet to be known, although policosanol was shown to inhibit cyclooxygenase enzyme activity [42] and was reported to lower the production of serum thromboxane [27, 43]. This explained why the extract used in the present study was able to inhibit AA-induced aggregation to a great extent. On the other hand, rice bran policosanol extract might enhance the production of cAMP by mediating adenylate cyclase activity via G_i_-coupled P_2_Y_12_ receptor modulation [32]. Moreover, rice bran policosanol extract might also inhibit collagen-induced platelet aggregation through direct binding to collagen, thus interfering with collagen-platelet interaction. Alternatively, rice bran extract may interact with the glycoprotein receptor, GPVI, to produce its effects. Reduced affinity of GPVI towards collagen attenuated thromboxane A_2_ synthesis through phospholipase C activity inhibition, and in addition, reduced degranulation and platelets recruitment [44, 45].

**Fig. 3:**
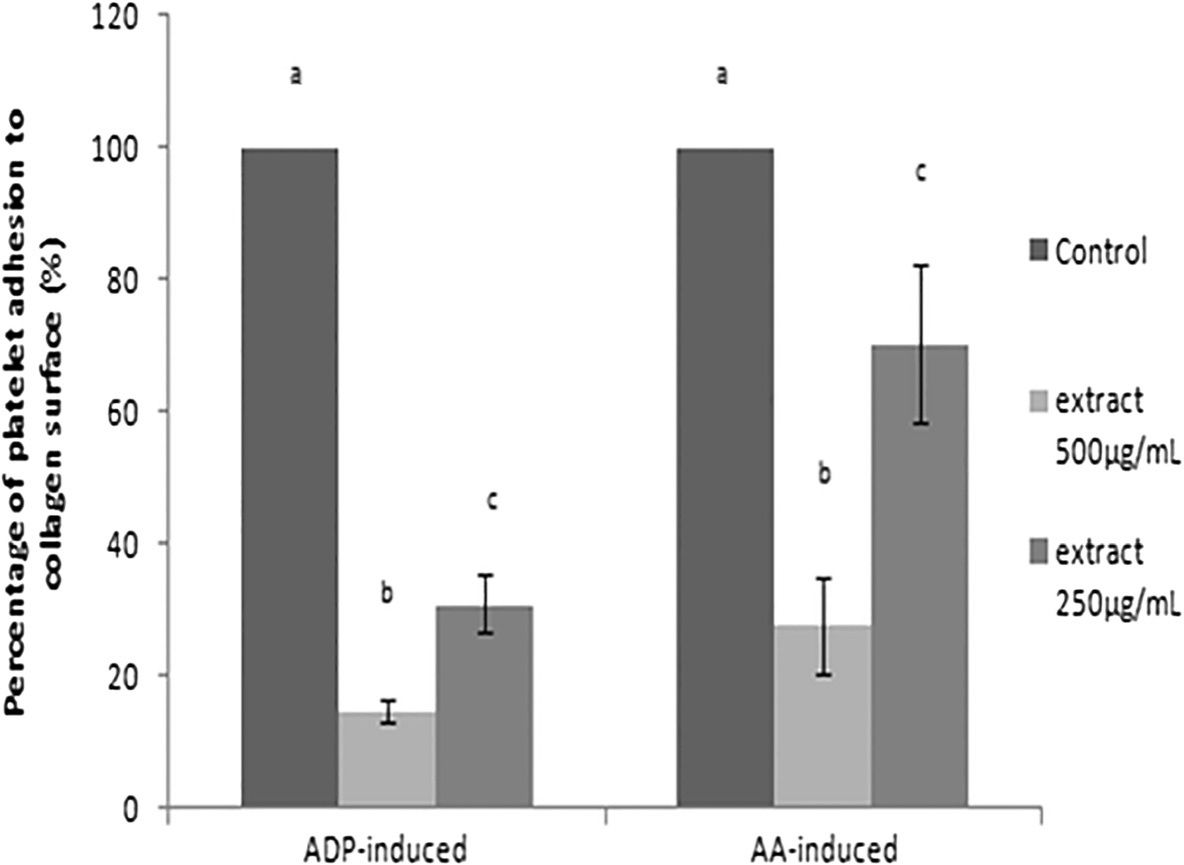
Platelet adhesion onto collagen-coated surface upon extracts treatment (in percentage after normalized with vehicle control). Means that shared the same letter were not significantly different (*p* < 0.05)

**Fig. 4:**
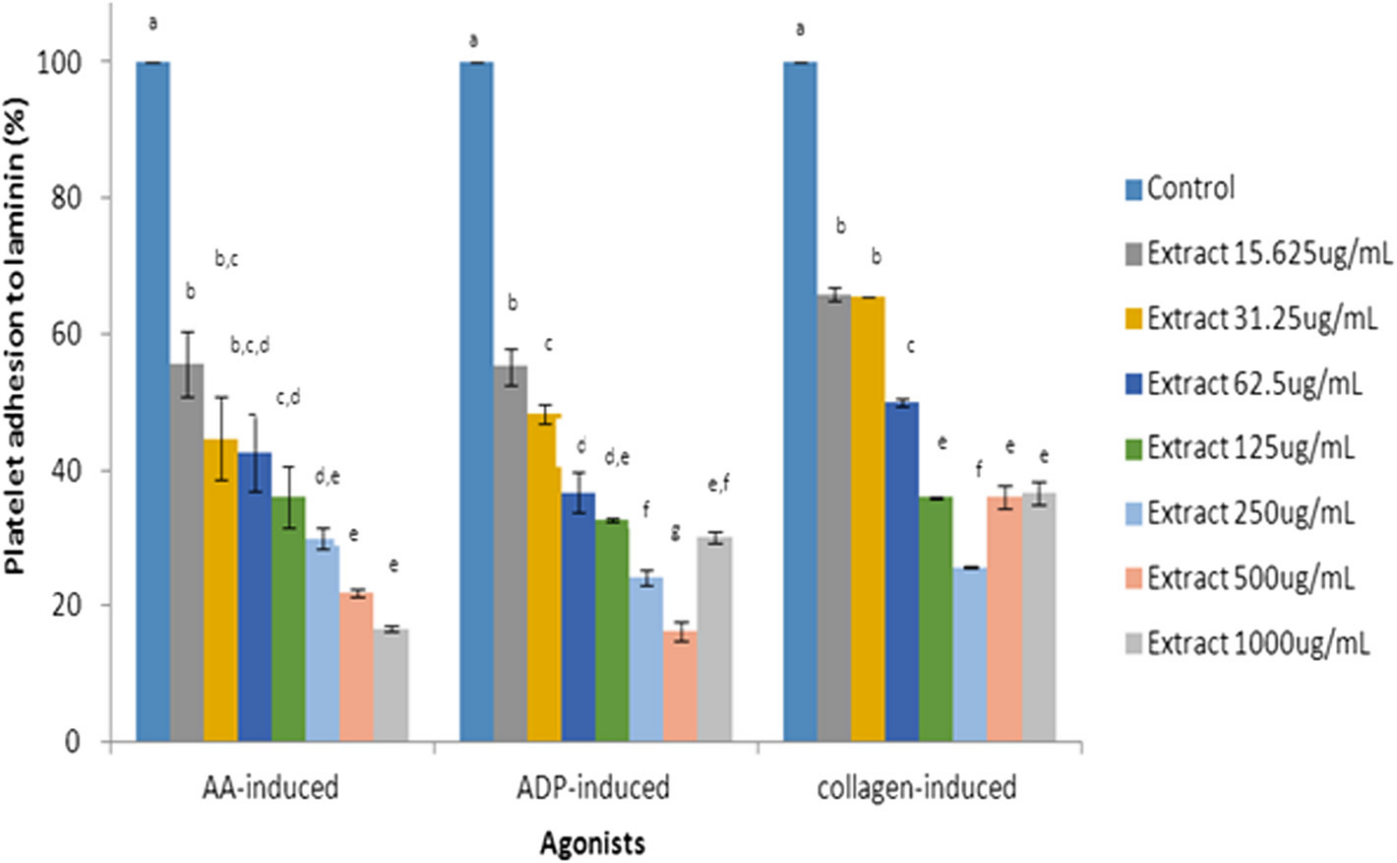
Platelet adhesion onto laminin-coated surface upon extracts treatment (in percentage after normalized with vehicle control. Means that shared the same letter were not significantly different (*p* < 0.05)

**Fig. 5:**
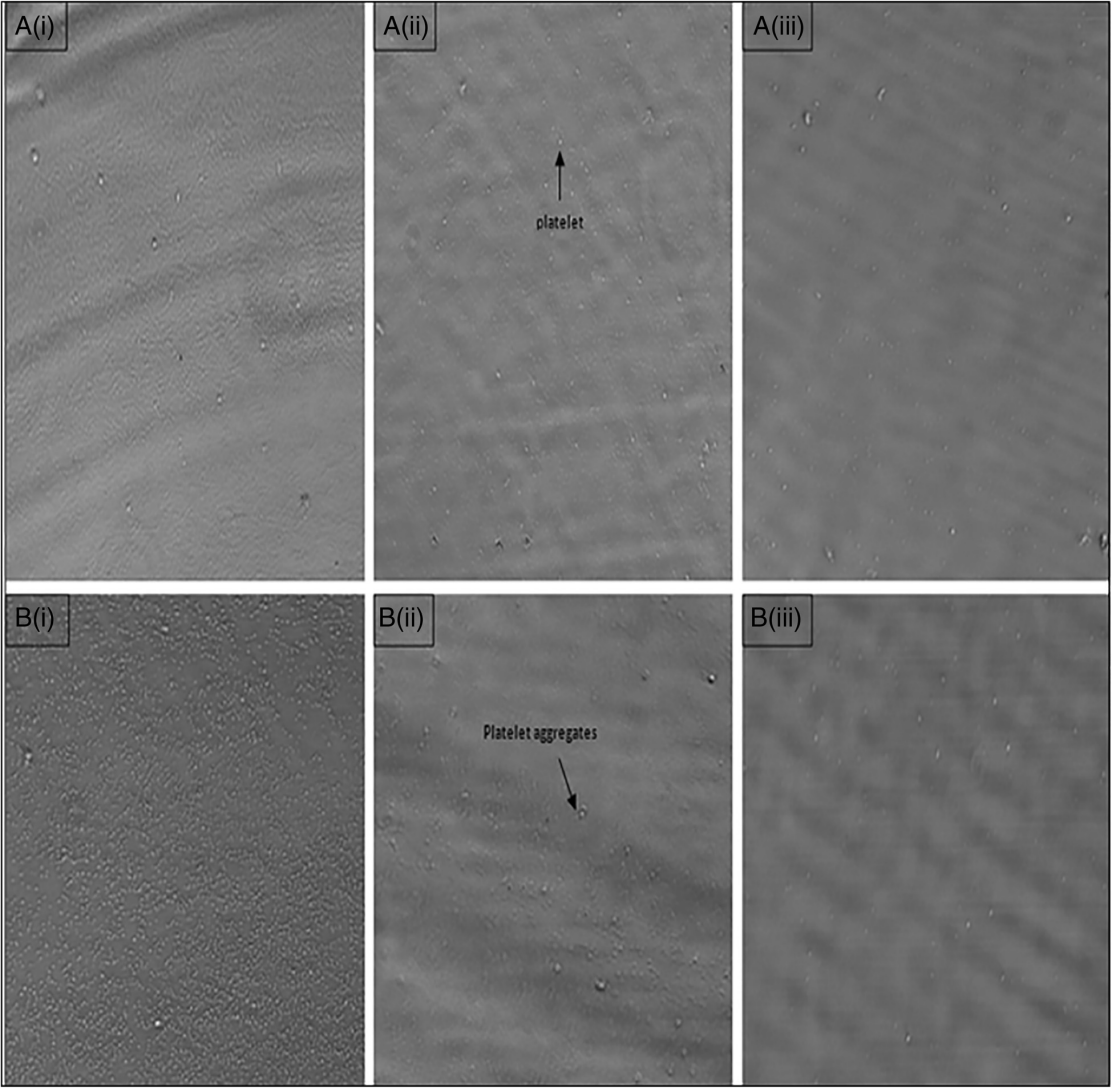
Microscopic images of platelet adhesion. Wells were coated with collagen. Platelets were pre-incubated with policosanol extract and activated with (**a**) AA (**b**) ADP. (i) Platelets without extract (DMSO control) (ii) Platelets incubated with 250 μg/mL policosanol extract (iii) Platelets incubated with 500 μg/mL policosanol extract. The wells were loaded with PBS after washing and examined under inverted microscope (×10)

Platelet suspension was pre-incubated with different concentrations of policosanol extract and the adhesive property of platelets onto laminin or collagen surface was examined. The extent of adhesion was studied using acid phosphatase assay, as shown on Figs. 3 and 4. Policosanol extract was shown to inhibit platelet adhesion onto collagen surface in a dose dependent manner. The crude policosanol extract at a low concentration of 12.5 μg/mL significantly inhibited platelet adhesion. At a concentration of 500 μg/mL, the extract significantly inhibited AA- and ADP-activated platelet adhesion by up to 72.32 ± 7.14 and 85.21 ± 1.90 %, respectively, while at 1000 μg/mL, it significantly inhibited AA-activated, ADP-activated and collagen-activated platelet adherence onto laminin surface by 83.39 ± 0.46, 69.86 ± 0.73 and 63.40 ± 1.74 %, respectively. The results showed that at 500 μg/mL, the extract was most effective in inhibiting ADP- and collagen-activated platelet adhesion to laminin, suggesting that it demonstrated a hormetic effect toward platelet adhesion whereby lower dosage was beneficial in certain responses, which is lost at higher dosages. Microscopic images of adhered platelets onto collagen-coated surface are shown in Fig. 5. The captured images provide semi-quantitative data in support of platelet modulation ability of policosanol extract. Compared to control depicted as Fig. 5a(i), policosanol extract significantly inhibited AA-activated platelets adhered onto collagen surface in a dose dependent manner. Likewise in Fig. 5b, the number of ADP-activated platelets adhered onto collagen surface was attenuated upon extract treatment.

**Fig. 6:**
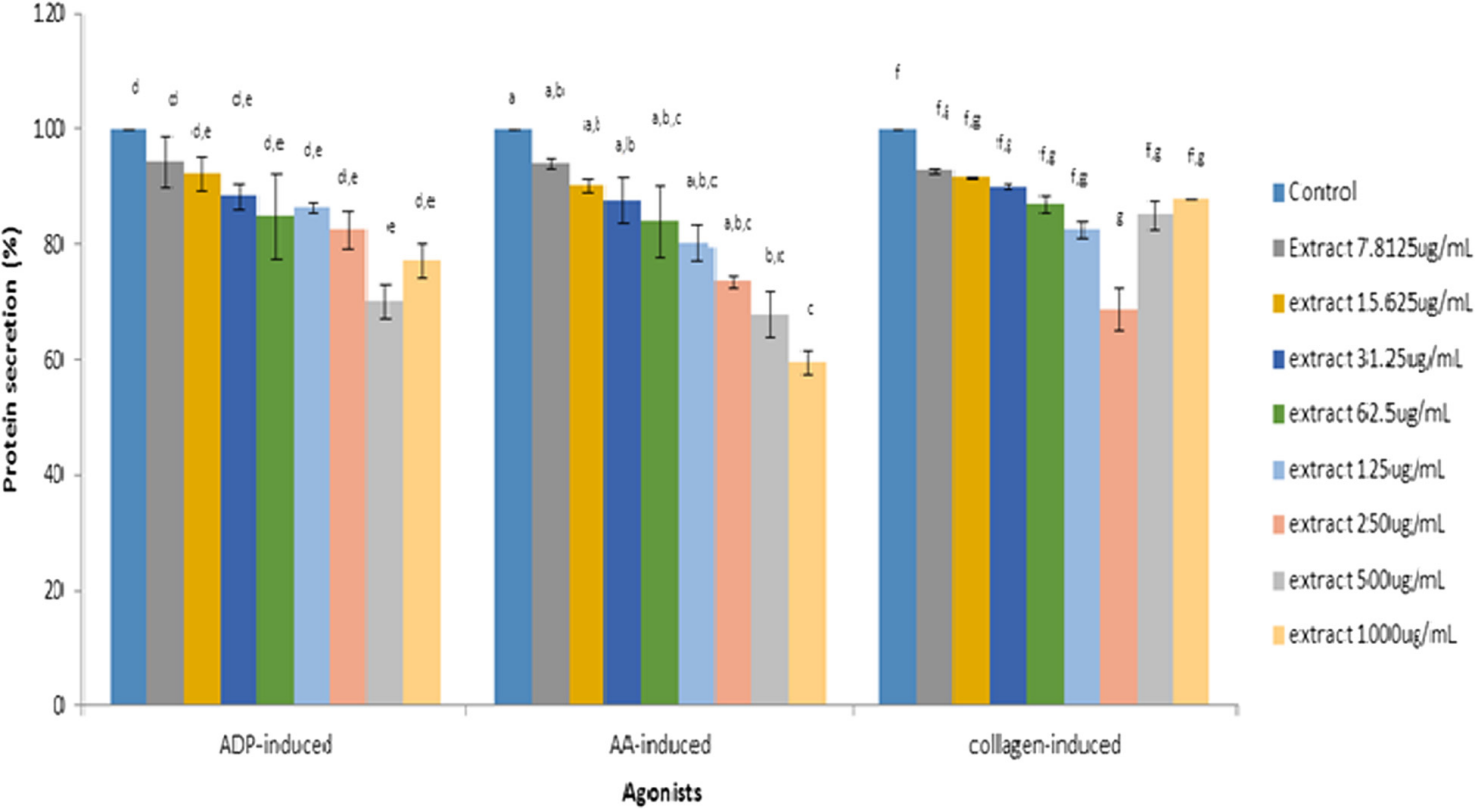
Cellular protein secretion determined by modified Lowry method upon extracts treatment (7.8125–1000 μg/mL) activated by ADP, AA and collagen. Protein concentration was determined using bovine serum albumin as standard (measurement wavelength at 650 nm). Means that shared the same letter were not significantly different (*p* < 0.05)

Crude rice bran policosanol extract might exert its aggregation inhibitory effect by targeting enzyme activities in the prostaglandin pathway. Impairment of prostaglandin synthesis pathway will reduce platelet adhesion, intracellular signaling and activation, and platelet-platelet interaction [46]. Policosanol extract could also possibly reduce the secretion of coagulating proteins, cell activating agents or adhesion molecules (e.g. platelet vWF, ADP) which are essential in platelet adhesion [37]. Figure 6 shows the attenuation of protein secreted from activated platelets. At a concentration of 500 μg/mL, the extract significantly attenuated protein secretion from ADP- and AA-activated platelets. However, the extract produced better inhibition of collagen-induced granular protein secretion at 250 μg/mL.

Glycoprotein expression is another possible target of policosanol action. Platelets possess receptors for various proteins such as collagen, fibronectin, fibrinogen, von Willebrand factor, laminin, thrombus protein and vitronectin [47]. Different responses of platelets towards different stimulants and compounds could imply inhibition of certain signaling molecules, pathways or specific receptors. Platelets bind to collagen directly or indirectly via GPIV, GPVI or GPIa/IIa, while their interaction with laminin is mediated by integrin GPIc/IIa (VLA-6) receptor [48]. Platelet studies have shown that initial platelet adhesion appears to be mediated by GPIIb/IIIa [49]. Any ligand binding or blockage to these glycoprotein receptors might probably inhibit platelet adhesion to collagen and laminin to a certain extent.

It is believed that policosanol extract can inhibit platelet hyperactivity through a multitude of mechanisms including scavenging of reactive oxygen species (ROS) [50]. Presence of other bioactive constituents in the crude extract, for instances, phenolics, aldehydes, or flavonoids might exert antioxidant ability synergistically, and thus prevent platelet adhesion. Signalling pathways during platelet activation, for instance metabolism of AA by COX and lipoxygenase, metabolism of phosphoinositide and glutathione cycle mediate intracellular ROS production [37]. Holistically, strong ROS scavenging ability can effectively attenuate platelet activation. To summarize, the present study provided a scientific basis to support consumption of rice bran in promoting good health, more specifically, in preventing cardiovascular diseases.

## Conclusion

The present study demonstrated that crude rice bran policosanol extract effectively inhibited platelet aggregation and platelet adhesion onto laminin and collagen surfaces, and attenuated protein secretion from platelets induced by different platelet activators. The present study provides further insights into the health value of rice bran. These findings serve as a scientific platform to further explore alternative therapies in cardiovascular diseases.

## Abbreviations

AA, Arachidonic acid; ADP, Adenosine diphosphate; BSA, Bovine serum albumin; COX, Cyclooxygenase; PBS, Phosphate buffer solution; PRP, Platelet rich plasma; ROS, Reactive oxygen species; vWF, von Willebrand factor
